# Biologic Therapies for Severe Asthma: Current Insights and Future Directions

**DOI:** 10.3390/jcm14093153

**Published:** 2025-05-02

**Authors:** Nuno Faria, Maria Inês Costa, Ana Luísa Fernandes, António Fernandes, Beatriz Fernandes, Daniela Cunha Machado, Francisco Machado, Laura Simão, Liliana Ribeiro, Lurdes Ferreira, Rita Boaventura, Ricardo Lima, Jorge Ferreira

**Affiliations:** 1 Pulmonology Department, Unidade Local de Saúde de Santo António, 4099-001 Porto, Portugal; 2 Instituto de Ciências Biomédicas Abel Salazar, University of Porto, 4050-313 Porto, Portugal; 3 Pulmonology Department, Unidade Local de Saúde de Matosinhos, 4464-513 Matosinhos, Portugal; 4 Pulmonology Department, Unidade Local de Saúde de Alto Ave, 4835-044 Guimarães, Portugal; 5 Pulmonology Department, Unidade Local de Saúde de Braga, 4710-243 Braga, Portugal; beatrizceleiros@gmail.com (B.F.);; 6 Pulmonology Department, Unidade Local de Saúde de Gaia e Espinho, 4434-502 Vila Nova de Gaia, Portugal; 7 Pulmonology Department, Unidade Local de Saúde de São João, 4200-319 Porto, Portugal; 8 Faculdade de Medicina da Universidade do Porto, University of Porto, 4099-002 Porto, Portugal; 9 Pulmonology Department, Unidade Local de Saúde do Tâmega e Sousa, 4560-136 Penafiel, Portugal; 10 Pulmonology Department, Unidade Local de Saúde de Trás-os-Montes e Alto Douro, 5400-279 Vila Real, Portugal; 11 Pulmonology Department, Unidade Local de Saúde de Entre Douro e Vouga, 4520-211 Santa Maria da Feira, Portugal

**Keywords:** severe asthma, difficult-to-treat asthma, asthma biomarkers, airway inflammation, type 2 asthma, non-type 2 asthma, biologic therapies, novel asthma treatments

## Abstract

Severe asthma is a subset of difficult-to-treat asthma that requires the verification of inhaler technique, the correction of modifiable risk factors, as well as diagnosis and comorbidity review. When severe asthma is suspected, patients should undergo proper phenotyping (T2-high or T2-low) and be referred to a specialized severe asthma clinic. The current biologics for severe asthma treatment include omalizumab (anti-IgE), mepolizumab and reslizumab (anti-IL-5), benralizumab (anti-IL-5 receptor), dupilumab (anti-IL-4/IL-13), and tezepelumab (anti-TSLP). The outcomes to evaluate are the reduction in systemic corticosteroid use, the reduction in exacerbations and healthcare use, and improvement in symptoms and lung function. Comorbidities should be carefully considered, and if possible, addressed with the same biologic. Dupilumab, mepolizumab, and omalizumab are also approved for chronic rhinosinusitis with nasal polyps (CRSwNP), the most common asthma comorbidity. There are currently several clinical trials on biologics for severe asthma. Depemokimab is an ultra-long-acting anti-IL-5 antibody with promising results in phase III trials as a twice-yearly biologic for T2-high asthma. Verekitug follows a similar dosing concept, targeting TSLP, but is still undergoing phase II trials. Itepekimab and astegolimab are two anti-IL-33 antibodies that could have a role in the future treatment of severe asthma. Tezepelumab is in a phase III clinical trial for CRSwNP. Besides new drugs, there is still a need for major research into biologics in severe asthma cases, namely with comparative studies, better biomarkers for predicting response, and the determination of optimal treatment duration.

## 1. Introduction

Asthma is a heterogeneous disease, characterized by variable airway inflammation, leading to symptoms such as wheezing, shortness of breath, chest tightness, and coughing, which fluctuate in both intensity and frequency over time. Affecting around 300 million people around the world, with a global prevalence of around 3.75%, asthma presents a significant burden on public health [1].

The primary cause of asthma-related deaths worldwide is the lack of access to inhaled corticosteroids (ICSs), which is particularly evident in low- and middle-income countries [2]. In high-income countries, the underuse of ICSs, along with delays in managing severe asthma are the leading causes of poor disease control. These factors heighten the risk of exacerbations, respiratory failure, and asthma-related mortality [3].

Difficult-to-treat asthma is defined as asthma that requires or remains uncontrolled under a medium- or high-dose ICS (Table 1) plus a second controller (usually long-acting β adrenoceptor agonists [LABAs]) to prevent it from becoming uncontrolled, or which needs maintenance with oral corticosteroids (OCSs) [1]. Uncontrolled asthma is, according to the ERS/ATS and the GEMA, characterized by two or more exacerbations requiring systemic glucocorticoids (or at least one hospitalization) in the last 12 months, persistent poor symptom control (Asthma Control Test [ACT] < 20 or Asthma Control Questionnaire [ACQ] ≥ 1.5), or airflow limitation (FEV1/FVC below the lower limit of normal or FEV_1_ < 80% predicted) [4,5]. According to the GINA, uncontrolled asthma is defined as poor symptom control (frequent symptoms or reliever use, activity limited by asthma, and night waking due to asthma) and/or frequent exacerbations (≥2/year) requiring an OCS or serious exacerbations (≥1/year) requiring hospitalization.

Severe asthma, accounting for 3.7% of all patients with asthma, is a subset of difficult-to-treat asthma that requires the confirmation of proper inhaler technique, the optimization of modifiable risk factors, and the thorough assessment of diagnosis and comorbidities. Therefore, in difficult-to-treat asthma, clinical history should be reassessed, lung function tests documenting variable flow obstruction should be obtained, as well as an hemogram (including eosinophils count), fractional exhaled nitric oxide (FeNO), total IgE, and serum/skin allergy tests. If differential diagnoses are suspected (e.g., chronic obstructive pulmonary disease, vocal cord dysfunction, and cardiac conditions), it is important to rule them out with necessary tests, such as a chest computed tomography (CT) scan, echocardiogram, and flexible bronchoscopy/laryngoscopy. After confirming an asthma diagnosis, it is important to rule out inadequate treatment (verify inhaler technique and assess for suboptimal ICS/LABA dosing), poor adherence, and uncontrolled comorbidities (e.g., obstructive sleep apnea, gastroesophageal reflux disease, hypertension, and atrial fibrillation) [1].

Non-adherence must be ruled out as a major cause of poor asthma control, exacerbating the factors addressed, and other potential contributing conditions must be assessed and excluded. In patients with a significant smoking history, the asthma diagnosis should be reassessed, considering the possibility of chronic obstructive pulmonary disease (COPD) as a comorbidity. Medical therapy should be scaled up to high-dose ICSs, long-acting β adrenoceptor agonists (LABAs), long-acting muscarinic antagonists (LAMAs), and leukotriene receptor antagonists (LTRAs) if not implemented before.

Physical activity and smoking cessation should be actively promoted. Immunization status should be reviewed based on local guidelines, including vaccinations for pneumococcal disease, pertussis, influenza, respiratory syncytial virus (RSV), and COVID-19. Regardless of the local guidelines, annual influenza vaccination is strongly recommended for patients with severe asthma. Specific immunotherapy (subcutaneous or sublingual) could be an option for allergic asthma, ideally with FEV1 greater than 70%.

Azithromycin (three times a week) may be considered for adults with persistent symptomatic asthma despite high-dose ICS-LABA. Before starting, sputum should be tested for nontuberculous mycobacteria, and ECG results should be checked for long QTc, with reassessment after a month. The risk of antibiotic resistance must be considered. At a dose of 500mg three times a week, diarrhea is a common side effect of azithromycin. A minimum six-month treatment is advised, as benefits were not evident at three months in clinical trials. The evidence from two trials suggests that azithromycin reduces asthma exacerbations in patients on medium- or high-dose ICS-LABA, regardless of their eosinophilic status. Due to the risk of antibiotic resistance, its use should only be considered after specialist consultation [1].

Bronchial thermoplasty may be considered for select patients with severe asthma, but its use is highly limited, and there are currently few centers performing it. While some studies indicate a reduction in exacerbations after the first three months of treatment, in patients reliant on high doses of ICSs, thermoplasty was associated with an initial increase in asthma exacerbations during the first three months and did not lead to improvements in lung function or symptomatic control [1].

After any of the above interventions, an appointment must be scheduled to assess patient’s response after at least 3 to 6 months. Asthma should be classified as severe only after addressing all the treatable traits. This approach ensures appropriate treatment, prevents unnecessary escalation, and enables targeted interventions such as biologic therapy for patients with severe asthma.

Besides a medical specialist, a comprehensive approach in an asthma clinic involving nurses, dietitians, physiotherapists, and psychologists experienced in asthma can significantly improve disease control and enhance the patient’s quality of life [6].

Considering that most local regulations demand maintenance OCSs or at least 2–3 exacerbations requiring OCSs before biologic therapy prescription, severe asthma is often accompanied by OCS side effects associated with substantial social and economic burdens [7,8]. The emergence of biologic therapy for severe asthma has been changing the landscape of the disease, with several drugs providing sustained disease control, mainly if at least a low-dose ICS is maintained [9].

Following severe asthma diagnosis, it is important to identify the biomarkers to infer our patient’s phenotype and biologic viability. Currently, the feasible biomarkers with a clinical impact are serum, sputum and biopsy eosinophil counts, total serum IgE, FeNO, and serum/skin allergy tests. These biomarkers enable the categorization of type 2 (T2 or T2-high) and non-type 2 (non-T2 or T2-low) inflammation, as well as either allergic or non-allergic. Elevated blood eosinophil counts (usually ≥150–300 cells/μL), high sputum eosinophil percentages (≥2–3%), and increased FeNO levels (≥25 ppb) are markers suggestive of an eosinophilic mechanism. Elevated total serum IgE and positive allergy tests (specific IgE or skin prick tests) are the markers suggestive of an allergic mechanism. All of these are markers of type 2 inflammation. Conversely, low blood and sputum eosinophil counts, FeNO levels below 25 ppb, and negative allergy testing support the diagnosis of non-type 2 inflammation, which is more often associated with neutrophilic or paucigranulocytic asthma. T2 inflammation is driven by a cascade produced by type 2 T-helper cells (T_H_2) and type 2 innate lymphoid cells (ILC2), promoting interleukins (IL) 5, 4, and 13, along with type E immunoglobulins. In recent years, the role of ILC2 in the pathogenesis of T2 asthma has been clarified with evidence of the triggering effect of thymic stromal lymphopoietin (TSLP) and IL-33, which have been correlated with more severe asthma, as well as glucocorticoid resistance [10]. Allergic asthma tends to present in childhood and be associated with atopy history and eosinophilic airway inflammation. This contrasts with non-allergic asthma, which is usually associated with eosinophilic, neutrophilic, or paucigranulocytic airway inflammation. Adult-onset asthma is usually eosinophilic and is more likely to evolve into severe asthma [5,11,12].

Upon confirming a severe asthma diagnosis, each patient should undergo proper phenotyping and be referred to a specialized consultant. The six biologics currently available enable the adequate treatment of most patients with severe asthma who do not respond to conventional therapy, with the primary challenge now being insufficient referral to specialized severe asthma clinics [13,14]. Their treatment should be tailored individually by an asthma specialist, ensuring that local regulations are followed if biological therapy is required.

As biologic therapies continue to evolve rapidly and the management of severe asthma grows increasingly complex, there is a growing need for a nuanced understanding of patient selection, biomarker-driven strategies, and real-world effectiveness. This review aims to provide a thorough and critical appraisal of the current biologic therapies for severe asthma, offering practical insights to guide clinical decision making in this dynamic field.

## 2. Biologic Therapies in Asthma

The current biologics for severe asthma treatment include omalizumab (anti-IgE), mepolizumab and reslizumab (anti-IL-5), benralizumab (anti-IL-5 receptor), dupilumab (anti-IL-4 receptor α, blocking both the IL-4 and IL-13 pathways), and tezepelumab (anti-TSLP). All of these are administered subcutaneously and are available as an auto-injector pen/prefilled syringe, except for reslizumab, which demands intravenous administration.

**Omalizumab** was first approved for severe asthma in 2003, making it the first biologic therapy for asthma. Omalizumab is a monoclonal antibody that selectively targets IgE, preventing its binding to basophils and mast cells, thereby inhibiting degranulation. As a result, omalizumab is particularly beneficial for patients with atopic asthma. However, since it does not interact directly with the effector cells, it is not suitable for use during asthma exacerbations [15].

Omalizumab is approved for patients aged 6 years or older with persistent moderate-to-severe asthma, a positive skin/serum test for at least one perennial allergen, and total serum IgE levels between 30 and 1500 UI/mL Omalizumab is also approved for severe chronic rhinosinusitis with nasal polyps (CRSwNP) in adults and for chronic spontaneous urticaria (CSU) in patients aged 12 years or older, as stated in Table 2. Omalizumab is also approved by the U.S. Food and Drug Administration (FDA), but not by the European Medicines Agency (EMA), for IgE-mediated food allergies in patients aged 1 year and older, although these patients should continue to avoid all foods they are allergic to [16].

The dosage of omalizumab must be adjusted based on total serum IgE levels and body weight, administered subcutaneously every 2 or 4 weeks. Body weight dose adjustment may also present a limitation for obese patients, particularly those over 90 kg.

The most common adverse effects of omalizumab include local injection site reactions (typically within the first hour), cardiovascular/cerebrovascular serious adverse events (<2%), thromboembolic events (<1%), and early- or late-stage anaphylaxis (<1%). Due to the risk of anaphylaxis, a doctor may prescribe an adrenaline auto-injector for the patient to carry for 24 h post-injection. Additionally, the development of autoantibodies has been reported in less than 1% of cases [17,18].

**Mepolizumab** is a monoclonal antibody that selectively targets IL-5 with high affinity, inhibiting the connection to its receptor and the cascade of eosinophilic airway inflammation. Mepolizumab is approved for patients aged 6 years or older with severe asthma, as well as serum eosinophils >150/µL. Mepolizumab seems effective even in patients with indication for omalizumab or that are uncontrolled despite receiving omalizumab [19]. This biologic is also approved for severe CRSwNP in adults, eosinophilic granulomatosis with polyangiitis (EGPA) in adults, and for hypereosinophilic syndrome (HES) in patients aged 12 years and older.

The mepolizumab standard dose is 100 mg every 4 weeks, except for children up to 11 years old, whose dose is adjusted to 40 mg every 4 weeks. For EGPA/HES, the dose is 300 mg administered as three 100 mg injections every 4 weeks. Several clinical trials have explored extending the dosing interval of mepolizumab to 6–8 weeks after one year of asthma control on the standard regimen, but currently there are limited data on these extended dosing interval regimens [20,21]. Mepolizumab’s effect on previously recruited eosinophils is limited, lacking evidence for its use during exacerbations [22].

The most common adverse effects of mepolizumab include headaches (up to 19%), local injection site reactions (typically within the first hour), myalgias (<6%), and early- or late-stage anaphylaxis (<1%). Additionally, the development of autoantibodies has been reported in less than 1% of cases. Herpes Zoster infection has been described (<1%), and immunization should be offered before mepolizumab initiation [23,24].

**Reslizumab** is a monoclonal antibody that also targets IL-5, but unlike mepolizumab, it is administered intravenously. Reslizumab is approved for patients aged 18 years old with severe asthma and serum eosinophils >400/µL.

Reslizumab is administered in a weight-adjusted dose, every 4 weeks, intravenously. The most common adverse effects of reslizumab include a transitory raise in creatinine-phosphokinase (up to 20%), the development of autoantibodies (<5%), and early- or late-stage anaphylaxis (<1%) [25].

**Benralizumab** is a monoclonal antibody that selectively targets the IL-5 receptor, inhibiting the connection of IL-5 to its receptor on both eosinophils and basophils, limiting the recruitment, differentiation, maturation, and activation of those cells. Benralizumab also targets the receptor of natural killer cells, macrophages, and neutrophils, promoting pro-apoptotic cytokines known to stimulate eosinophil apoptosis.

Benralizumab is approved for patients aged 18 years or older with severe asthma, as well as serum eosinophils >300/µL. The benralizumab dose for severe asthma is 30 mg every 4 weeks for the first three administrations, followed by an 8-week interval between injections. Benralizumab is also approved for use in adults with EGPA at a dose of 30 mg every 4 weeks.

The most common adverse effects of benralizumab include the development of autoantibodies (up to 13%), headaches (8%), pharyngitis (3–4%), fever (3%), and hypersensitivity reactions (local site reactions, urticaria, angioedema, and anaphylaxis) that usually occur following injection (<3%), but can present later [26].

**Dupilumab** is a monoclonal antibody that selectively targets the IL-4 receptor, inhibiting the inflammatory cascade induced by IL-4 and its reaction to IL-13, also produced by T_H_2 lymphocytes after IL-4 receptor connection. This immune cascade blockage inhibits the IgM-to-IgE-class switch (limiting mastocytes and eosinophils activity), mucus production, and the production of pro-inflammatory cytokines that induce airway smooth muscle remodeling.

Dupilumab is approved for patients aged 6 years or older with severe asthma, as well as serum eosinophils ≥150/µL and/or FeNO ≥ 25. Dupilumab is also approved for uncontrolled COPD in adults with blood eosinophils ≥300/µL, severe CRSwNP in adults, moderate-to-severe atopic dermatitis in those ≥6 months-old, prurigo nodularis in adults, and eosinophilic esophagitis in those ≥12 years old. The dupilumab standard dose (if >17 years or >60 Kg) for severe asthma is the first 600 mg dose, followed by 300 mg every 2 weeks. For patients between 12 and 17 years old or <60 Kg, a first dose of 400 mg, followed by 200 mg every 2 weeks is recommended.

The most common adverse effects of dupilumab include head/neck dermatitis (up to 19.5%), local injection reaction (10%), transitory eosinophilia (up to 10%; patients with blood eosinophils >1500/µL are at higher risk of HES with dupilumab), and autoantibody development [27,28].

**Tezepelumab** is a monoclonal antibody targeting TSLP, preventing interaction with its receptor. Considering its upstream inhibitory activity on the inflammatory cascade, tezepelumab inhibits the TSLP-mediated activation of dendritic cells and ILC2 cells (primarily involved in type 2 asthma), T_h_17 cells (primarily involved in neutrophilic asthma), as well as mast cells, smooth muscle cells, and fibroblasts in paucigranulocytic asthma. Therefore, tezepelumab is able to reduce the level of several biomarkers, such as peripheral blood eosinophils, airway mucosa eosinophils, IgE, FeNO, IL-5, and IL-13.

Tezepelumab is approved for patients aged 12 years or older with severe asthma, regardless of their serum eosinophils, IgE, and FeNO levels. Of note, although it is approved for non–type 2 patients, tezepelumab is particularly effective in severe type 2 asthma [29]. The tezepelumab dose is 210 mg every 4 weeks subcutaneously.

The most common adverse effects of tezepelumab include pharyngitis (4.1%), arthralgia (3.8%), back pain (3.8%), and injection site reactions (3.8%). Although post-marketing surveillance studies observed a higher incidence of major adverse cardiovascular events in patients under tezepelumab, the European Medicines Agency (EMA) stated that no causal relationship has been established, nor has a population at risk been identified. However, the EMA recommends that patients should be advised of the symptoms suggestive of a cardiac event to prompt seeking immediate medical attention [30,31,32].

Table 3 summarizes the prescription criteria for the biologics mentioned above. Before prescribing a biologic for severe asthma, national regulations should be reviewed. Additionally, parasitic infections should be excluded through fecal culture or serological tests, especially in countries with high rates of helminthic infections [33]. It is also important to note that recent systemic corticosteroid therapy can affect the eosinophil levels in peripheral blood. In fact, a 5-day course of oral corticosteroids decreases the level of eosinophils by about 71%, and its effect may remain even 1-3 weeks after that short course [34]. In these cases, the patient’s history of eosinophilia should be carefully reviewed, as reduced eosinophil levels may reflect the suppressive effects of recent corticosteroid therapy rather than the patient’s true baseline.

The prescription of a biologic in severe asthma cases is not always an easy decision. The clinical characteristics and patients’ biomarkers should be reviewed. Comorbidities should be carefully considered, and if possible, addressed with the same biologic (Table 2). An elevated FeNO or high peripheral eosinophilia level is a good predictor of the response to the currently available biologics. The fact that a patient developed asthma only in adulthood may be a good marker of the response to anti-IL5 therapies [35]. However, there is currently no definitive evidence that one biologic is universally superior to the others. Even among the anti-IL-5/IL-5R biologics, there is no evidence of clear superiority between the three available drugs or between these and dupilumab or omalizumab [36,37,38,39]. This highlights the complexity of selecting a biologic for severe asthma, where decisions should be made on a case-by-case basis and in a specialized severe asthma clinic.

A minimum period from 4 to 6 months is required to assess the effectiveness of treatment with a biologic in severe asthma cases. The time for first response is variable and dependent on individual variability, the baseline biomarkers, and treatment adherence. For most biologics, the time to the first appearance of a clinical/functional benefit can be as short as 1–3 months [40,41]. Also, the maximal effect is usually noticed after 12–24 months for most biologics [42]. The outcomes we need to evaluate are the reduction in exacerbation, systemic corticosteroid use and healthcare use, as well as improvement or stabilization in lung function and symptoms [43]. A poor treatment response can result from non-compliance, uncontrolled comorbidities, the formation of autoantibodies, or the misinterpretation of a patient’s phenotype. Clinical remission (e.g., no asthma symptoms or exacerbations for a specific period) and complete remission (e.g., including normal lung function, airway responsiveness, and/or inflammatory biomarkers) have been increasingly explored in the literature as the ultimate goals of asthma treatment [1]. Clinical remission has emerged as an important treatment goal in severe asthma cases, but there is an urgent need for a better consensus-driven definition of clinical remission as it varies substantially between studies [44].

Currently, no guidelines recommend dual biologic therapy for severe asthma due to insufficient data and high costs. However, some observational studies with a small number of patients showed promising results without significant adverse events [45]. Also, giving azithromycin to patients with asthma taking biologics and experiencing chronic bronchitis and/or frequent purulent exacerbations may result in reduced annual rates of exacerbations and improved symptom scores [46]. Combining biologics (particularly omalizumab and tezepelumab) and allergen-specific immunotherapy has promising prospects in the clinical treatment of allergic rhinitis and asthma due to the improvements in both clinical efficacy and safety [47].

Biologic agent administration for severe asthma exhibits an excellent safety profile. These agents not only do not increase the occurrence of adverse events, but are actually associated with lower incidences of reported adverse events, serious adverse events, death, and pneumonia compared to the standard care treatments [48].

Finally, biologic therapy is an additional treatment for severe asthma, not a replacement for standard asthma medications. Patients should continue to use their prescribed inhalers even after starting biologics. If asthma is well controlled under biologic therapy, the OCS dose should be gradually decreased/ceased, checking for adrenal insufficiency. If asthma remains controlled afterwards, the add-on therapy (LAMA and LTRA) can be discontinued, followed by a decrease in the ICS dose. Patients with asthma on biologics should never stop taking a low-dose ICS [49].

### 2.1. Self-Administration of Biologics at Home

The home administration of biologics has greatly enhanced accessibility for patients with severe asthma. By eliminating the need for frequent visits to healthcare facilities, patients can receive their treatments in the comfort of their own homes, which is particularly beneficial for those who may have difficulty traveling or live far from medical centers. The self-administration of biologics at home can potentially decrease the burden on healthcare systems. Also, patients generally prefer the at-home administration of subcutaneous biologics, empowering patients to take a more active role in their asthma management, which leads to better adherence to treatment regimens and improved overall disease control [50].

However, home administration comes with an initial learning curve, with the potential for administration errors and the loss of regular clinical monitoring (including side effect management) [51]. Proper training and ongoing support from healthcare providers are crucial to mitigate this risk. The authors recommend that at least the first three administrations take place in a hospital setting, ensuring proper patient training for self-administration, while home administration is planned. Additionally, some authors advise that patients receiving biologic therapy have a medical appointment at least every three months, with the biologic administered at the hospital at the time of those visits to check their administration technique.

### 2.2. Pediatric Severe Asthma

In recent years, we have seen significant advances in the use of biologic therapies for managing severe asthma in children and adolescents. While omalizumab, mepolizumab, and dupilumab are the current mainstays for children aged 6 and above, benralizumab and tezepelumab expand the options for adolescents aged at least 12 years old. Currently, the HORIZON clinical trial (NCT06023589) is evaluating the potential use of tezepelumab in children aged 5–12. The use of biologics in pediatric patients with severe asthma involves several challenges. Treatment adherence may be compromised by children’s perception of how unpleasant/painful subcutaneous injections are. Also, children may not fully understand the significance of receiving treatment on a regular basis, highlighting the need for a strong caregiver support. Although some biological therapy trials demonstrate rare serious adverse events, the safety considerations remain paramount in pediatric populations. In children, continuous monitoring is essential, as their developing immune systems and unique physiological characteristics may influence the treatment responses [52].

As in adults, both the duration of treatment and the selection of the optimal biologic remain subjects of debate in the pediatric population. The rationale for biological initiation is similar to that of adults. Most pediatric severe asthma cases exhibit T2-high inflammation, characterized by elevated eosinophils, IgE, and FeNO levels [53]. The management of patients aged from 6 to 12 years old requiring biologic is limited by the availability of only three therapies. In cases with evidence of perennial allergy and IgE 30–1500 IU/mL, omalizumab may be considered. Omalizumab has evidenced long-term safety in pediatric asthma cases, with no anaphylaxis or oncological pathologies being reported in a 4-year prospective cohort [54]. Alternatively, mepolizumab and dupilumab are both suitable if serum eosinophils >1500/µL, the latter being particularly effective if FeNO > 20 ppm. Dupilumab is also preferred for children with comorbid atopic skin conditions. Mepolizumab use is independent of FeNO levels, and mepolizumab should be used if there is evidence of a clear eosinophilic phenotype without significant atopy, a history of multiple eosinophilic exacerbations (particularly if associated with viral triggers and high eosinophil levels), or a history of conjunctivitis/ocular disease (which is more common with dupilumab) [55].

No biologics are currently approved for children under 6 years due to limited clinical trials and diagnostic challenges. Differentiating severe asthma from other wheezing disorders (e.g., viral-induced wheeze) is difficult in preschoolers. Also, the immune mechanisms in early childhood asthma may differ from those of older children, requiring tailored therapeutic targets [52]. However, trials like LIBERTY ASTHMA TREKIDS (NCT06191315) for dupilumab in children aged 2–6 years and PARK (NCT02570984) for omalizumab in children aged 2–4 years will enhance our understanding of biologic use in this age group.

## 3. Future of Biologic Therapy for Asthma

There are currently several phase II and III clinical trials on biologics for severe asthma.

Depemokimab (ultra-long-acting anti-IL-5) is showing promising results in the treatment of severe asthma with an eosinophilic phenotype. Depemokimab is administered via subcutaneous injection every six months (twice yearly), which may improve treatment adherence and reduce healthcare utilization in patients with type 2 asthma as it presents binding affinity and high potency for IL-5. The safety profile of depemokimab appears favorable, with adverse events comparable to those of a placebo. While depemokimab shows promise in reducing exacerbations in phase III trials (SWIFT), further research and real-world data will help determine its long-term efficacy and place in asthma management [56].

Itepekimab (anti-IL-33) is a monoclonal antibody that is administered subcutaneously at a dose of 300 mg every 2 weeks, with proved benefits for asthma control, quality of life, and lung function [57]. Currently, it is being primarily investigated for COPD in two phase III clinical trials (AERIFY-1, which includes only former smokers, and AERIFY-2), with the results expected to be produced in the coming year [58].

Tozorakimab (anti-IL-33) is a monoclonal antibody studied in phase IIa clinical trial (FRONTIER-3) that is administered subcutaneously at a dose of 600 mg every 4 weeks, with potential benefits for early-onset eosinophilic (>300/µL) moderate-to-severe asthma [59].

Astegolimab (anti-ST2 receptor), a human IgG2 monoclonal antibody that also selectively inhibits the IL-33 receptor ST2, has shown promising results in the treatment of severe asthma, particularly for patients with low blood eosinophil counts. In a 4-week dosing regimen, astegolimab reduced exacerbations even in patients with low eosinophil levels, considered as <300/µL, in a phase IIb trial (ZENYATTA) [60]. However, this phase IIb trial was published in 2021, and no phase III trial has been registered to date.

While tezepelumab has been primarily studied for severe asthma, its beneficial effect on CRSwNP has already been evidenced in a clinical trial (phase III WAYPOINT trial), with significantly greater reductions in the size of nasal polyps, the severity of nasal congestion and sinonasal symptoms, as well as the need for surgery or glucocorticoids [61]. For COPD, a phase IIb trial (COURSE trial) showed a 17% reduction in COPD exacerbations with tezepelumab compared to those of a placebo, with a greater effect on those with higher eosinophil counts, but no phase III trials have been registered yet [62].

Verekitug (anti-TSLP) is a humanized anti-TSLP receptor monoclonal antibody that has higher binding affinity and potency than tezepelumab, which may allow for less frequent dosing up to every six months (as opposed to tezepelumab monthly posology). There are two phase II clinical trials actively administering verekitug to patients with severe asthma and CRSwNO (VIBRANT and VALIANT) [63].

Ecleralimab (anti-TSLP) is a novel inhaled anti-TSLP that in a phase IIa proof-of-concept study, was well tolerated and reduced allergen-induced bronchoconstriction in adults with mild asthma. The inhaled delivery method of ecleralimab may offer advantages over the current injectable biologic therapies by allowing for the direct targeting of the lungs with potentially reduced systemic effects. This novel approach represents a promising new therapeutic class for asthma treatment [64].

Lunsekimig (anti-IL-13/anti-TSLP) is a nanobody that blocks both IL-13 and TSLP and is currently included in a phase II clinical trial for moderate-to-severe asthma (AIRCULES) [63].

Mepolizumab is at a later stage of research for COPD (phase III MATINEE trial), presenting significant reduction in moderate-to-severe exacerbations in patients with type 2 inflammation (defined as eosinophil count ≥300/µL). Mepolizumab use in COPD is currently being reviewed for eventual approval by the FDA.

Several monoclonal antibodies have been previously studied for severe asthma, yet failed to demonstrate any advantage over those already available. Tralokinumab is a human recombinant monoclonal antibody that binds IL-13, blocking its connection to the IL-13 receptor. Tralokinumab use has been studied for subcutaneous administration at doses of 300mg every 2 weeks. However, its inconsistent results in the STRATOS 2 clinical trial do not support a key role for this anti-IL-13 antibody in severe asthma [65]. Similar results have been seen with another anti-IL-13 antibody, lebrikizumab. The other antibodies that showed initial promise, but were ultimately discontinued in phase II clinical trials were those targeting IL-9 (enokizumab) and IL-17A (secukinumab) [66].

The ABRA trial tested benralizumab (anti-IL5Rα) during active eosinophilic exacerbations of asthma/COPD and evidenced a reduced failure rate (against prednisolone) and reduced hospital readmissions [67]. This is the first trial demonstrating acute biologic efficacy during exacerbations rather than prevention.

Mucus plugging in asthma is linked to increased disease severity, frequent exacerbations, and diminished lung function. Multidetector computed tomography (CT) has emerged as a noninvasive tool for assessing airway mucus accumulation. In severe asthma, mucus may define a distinct clinical phenotype that contributes to airway obstruction and impaired ventilation, offering a potential target for therapy. The recent studies on benralizumab, dupilumab, tezepelumab, and mepolizumab have explored this approach [68].

The discontinuation of biologic therapies in severe asthma cases has been a topic of increasing interest in recent years and is closely linked to the concept of clinical remission [69]. Several factors have been identified as potential predictors of successful biologic discontinuation, including lower peripheral eosinophil counts during treatment, suppressed T2 inflammation (evidenced by lower blood eosinophil counts and/or FeNO levels), the absence of significant asthma symptoms, normalized pulmonary function, and the control of asthma comorbidities. While some patients maintain asthma control after stopping biologics, others may experience the worsening of symptoms and a higher risk of exacerbations [70,71]. The data from the COMET study revealed an increase in exacerbations and reduced asthma control after mepolizumab discontinuation [72]. Research on omalizumab demonstrated similar results [73]. For dupilumab, a clinical trial (WIDUSA, NCT06818019) has just started to investigate if discontinuation is feasible. The OPTIMAL algorithm provides a potential framework for the safe down-titration of biologics in well-controlled patients with severe asthma [21].

Besides new drugs, there is still a need for major research into biologics in severe asthma cases, namely comparative studies (11% of patients need to switch from their initial biologic) on the biomarkers for predicting response and monitoring, defining treatment duration, and assessing the risk of immunogenicity more precisely [68].

## 4. Conclusions

The advent of biologic therapies has revolutionized the treatment landscape for severe asthma. However, before considering these advanced therapies, it is crucial to thoroughly review asthma diagnoses, assess for inadequate treatment, and address comorbidities. This comprehensive evaluation ensures that patients are not misclassified as having severe asthma when other factors may be contributing to poor symptom control. Implementing add-on therapies such as LAMA or LTRA should be considered before stepping up to biologic treatments. Also, a patient with severe asthma should always be referred to a severe asthma clinic.

The future of biologic therapy in asthma management is likely to focus on optimizing treatment regimens. Studies investigating the potential for dose tapering or extending dosing intervals will be crucial in balancing efficacy with cost-effectiveness. An important limitation in the current evidence base for biologic therapies in severe asthma cases is the scarcity of head-to-head comparative studies. Most available data arise from individual randomized controlled trials with differing inclusion criteria, endpoints, and study designs, or from observational real-world studies. This heterogeneity makes direct comparisons between biologics challenging and requires careful interpretation when extrapolating the results to clinical practice. Additionally, the development of new molecules with improved efficacy or the broader targeting of inflammatory pathways may further enhance the therapeutic options available to clinicians. As biologic therapies become more affordable and accessible, their potential use in emergency settings could represent a paradigm shift in acute asthma management. The rapid onset of action and targeted approach of these therapies could potentially reduce the hospitalization rates and improve the outcomes for patients with severe exacerbations.

In conclusion, while biologic therapies offer promising options for patients with severe asthma, their use should be preceded by the thorough assessment of the patient’s condition and the exploration of other add-on therapies. Ongoing research into optimizing biologic treatments and their potential expanded applications underscores the dynamic nature of asthma management and the importance of continued innovation in this field.

## Figures and Tables

**Table 1:** Definition of medium and high daily doses of inhaled corticosteroids according to GINA.

Inhaled Corticosteroid	≥12 Years Old		6–11 Years Old	
	High Dose	Medium Dose	High Dose	Medium Dose
Beclomethasone (standard)	>1000	>500	>400	>200
Beclomethasone (extrafine)	>400	>200	>200	>100
Budesonide (MDI or DPI)	>800	>400	>400	>200
Ciclesonide	>320	>160	>160	>80
Fluticasone propionate (MDI or DPI)	>500	>250	>200	>100
Fluticasone furoate (DPI)	200	100 *	N.A.	50 *
Mometasone MDI	>400	>200 *	200	100 *
Mometasone DPI (Breezhaler triple/Breezhaler double/Twisthaler)	160/320/800	80/160/400	N.A. ^#^	N.A. ^#^

Doses in µg. * Medium and low doses are same for these inhaled corticosteroids. ^#^ Mometasone Twisthaler is available in 110 μg dose for those between 6 and 11 years old. DPI: dry powder inhaler; MDI: metered-dose inhaler; N.A: not available.

**Table 2:** Currently approved indications for biologics used by those with severe asthma.

	Omalizumab	Mepolizumab	Benralizumab	Reslizumab	Dupilumab	Tezepelumab
Indications	CRSwNP	CRSwNP	EGPA	-	CRSwNP	-
beyond severe	CSU	EGPA			COPD	
asthma	Food allergy *	HES			Atopic dermatitis	
					Prurigo nodularis	
					Eosinophilic esophagitis	

* Approved for food allergies by FDA, but currently not by EMA. COPD: chronic obstructive pulmonary disease; CRSwNP: chronic rhinosinusitis with nasal polyps; CSU: chronic spontaneous urticaria; EGPA: eosinophilic granulomatosis with polyangiitis; HES: hypereosinophilic syndrome. Note: All these biologics are approved for severe asthma.

**Table 3:** Biologics available for severe asthma.

	Omalizumab	Mepolizumab	Benralizumab	Reslizumab	Dupilumab	Tezepelumab
Target	IgE	IL-5	IL-5R	IL-5	IL-4/13	TSLP
Age	≥6 years	≥6 years	≥12 years	≥18 years	≥6 years	≥12 years
Eosinophils	-	≥150	≥00	≥400	≥150	-
IgE	30 a 1500	-	-	-	-	-
Administration	SC	SC	SC	IV	SC	SC
Dosing interval	2 or 4 weeks	4 weeks	8 weeks *	4 weeks	2 weeks	4 weeks

IV: intravenous; SC: subcutaneous. * First, second, and third administrations of benralizumab must be administered every 4 weeks, subsequently increasing dosing interval to every 8 weeks.
